# Shooting the messenger: Ecosystem approaches in haematology

**DOI:** 10.1002/hem3.70444

**Published:** 2026-08-03

**Authors:** Stephen P. Hibbs

**Affiliations:** ^1^ Wolfson Institute of Population Health Queen Mary University of London London United Kingdom

Treatment breakthroughs often take indirect pathways. Instead of targeting the affected cell or gene, bispecific antibodies redirect endogenous T‐cells towards malignant B‐cell lines, and gene therapy for sickle cell disease drastically upregulates production of fetal hemoglobin. Some cancer treatments act on non‐human intermediaries: antibiotics against *Helicobacter pylori* or direct‐acting antivirals against the hepatitis C virus can induce remissions in some cases of marginal zone lymphoma.^1^

This article surveys two emerging treatment approaches directed to contrasting haematological clinical problems—dengue haemorrhagic fever and graft‐vs‐host‐disease (GvHD)—acting on the wider ecosystems in which these conditions emerge. Beyond discovering novel treatment options, understanding haematological conditions through an ecological lens will become indispensable as the human‐induced climate crisis unfolds.

## ADDRESSING DENGUE THROUGH A MOSQUITO SEXUALLY TRANSMITTED INFECTION

Could an arthropod sexually transmitted infection be part of the treatment arsenal for a disease of haematological significance? *Aedes aegypti* is the mosquito vector for viral diseases including dengue, Zika, chikungunya, and yellow fever. Each of these viruses can cause haematological complications; dengue can cause profound thrombocytopenia, coagulopathy, and bleeding complications. For decades, dengue transmission has been disrupted through insecticides and repellants, but these strategies are only partially effective.

A counterintuitive approach is to reduce the viability of *A. aegypti* by infecting mosquitoes with *Wolbachia pipientis*—sexually transmitted bacteria—which reduces the viability of mosquito offspring. There are many ways to deploy this technique, and one of the most effective is known as the incompatible insect technique–sterile insect technique (IIT‐SIT). IIT‐SIT involves sex‐selecting male mosquitoes, infecting these mosquitoes with *W. pipientis*, and finally irradiating to ensure any mis‐sorted female mosquitoes are infertile. These infected mosquitoes are released to breed with wild‐type females, but due to incompatibility between Wolbachia‐infected and ‐uninfected mosquitoes, the offspring are not viable. This approach has not previously been trialed at scale.^2^

Lim et al. conducted a cluster randomised trial of IIT‐SIT to test whether this approach could reduce dengue incidence.^2^ The trial took place across regions of Singapore in which over 10% of the population live. In clusters receiving repeated IIT‐SIT releases over a 2‐year period, the *A. aegypti* mosquito population fell almost sevenfold compared to control clusters. More importantly, residents living within intervention clusters were 70% less likely to contract symptomatic dengue virus.^2^ If dengue complications such as coagulopathy and thrombocytopenia are also reduced—a likely but unreported result of this intervention—then IIT‐SIT represents a highly unexpected route to preventing a disease of haematological significance.

## THE GUT MICROBIOME

Human bodies are ecosystems in themselves, providing a home for a vast array of microbial species. Across recent decades, the role of the gut microbiome has been established across many diseases. Research presented at the European Hematology Association (EHA) congress this year alone demonstrates the microbiotal impact on pathogenesis, severity, or treatment of a wide variety of haematological conditions or treatments: chronic myeloid leukaemia (CML), acute myeloid leukaemia (AML), aplastic anemia, myeloma, diffuse large B‐cell lymphoma (DLBCL), follicular lymphoma, chronic lymphocytic leukaemia (CLL), allogeneic stem cell transplant, and sickle cell disease.^3^, ^4^

GvHD illuminates the complexity of the gut microbiotal ecosystem in haematological disease. The gut microbiota faces multiple insults during allogeneic transplant, including conditioning regimens, antibiotics, and reduced oral nutritional intake. This leads to a reduction in microbiotal diversity—dysbiosis—leading to dominance of harmful bacteria, disruption of gut epithelial barriers, and alteration of the gut immune microenvironment.^5^

There is a further layer of complexity: in an allograft context, donors are donating more than their hematopoietic stem cells alone. Following an allogeneic transplant, gut microbiota originate from two different bodies: the hematopoietic transplant itself is not sterile, and donor microbial transfer makes a significant contribution to microbiotal reconstitution post‐transplant.^6^ Furthermore, the diet of a recipient before and after transplantation influences both the risk and outcome of GvHD by selectively altering the constitution of the gut microbiome.^7^ Gut GvHD can be understood as a dynamic interplay between host and donor microbiota, donor lymphocytes, recipient gut epithelium, and nutrition.

Faecal microbiota transplantation (FMT) and probiotic strategies have shown promising but variable efficacy on GvHD in small studies.^6^ Given the intricacy of the post‐transplant gut ecosystem, it may take time to identify an elegant and effective microbiotal therapeutic intervention.

## ECOSYSTEM APPROACHES IN A TIME OF PLANETARY UPHEAVAL

These two examples show the value of thinking ecologically: considering the contribution of non‐human species to haematological conditions to spark new therapeutic approaches. One such approach has already been successfully implemented at scale: a 2024 meta‐analysis demonstrated that every 1% increase in population usage of insecticide‐treated bed nets in sub‐Saharan Africa resulted in a 2% reduction in childhood Burkitt lymphoma incidence.^8^

Beyond treatment discovery, there is a further urgent rationale to adopt an ecological lens in haematology: human‐induced climate change.^9^ Some impacts are intuitive, such as new haemovigilance challenges as the epidemiology of blood‐borne disease vectors changes with climate.^10^ Other sequelae have indirect but profound impacts across haematological practice, such as the ways that the human microbiome is changing as a result of climate change.^11^ These mechanisms, and myriad other ways climate change is changing our world, may augment every characteristic of haematological conditions and treatment. Like other sequelae of the climate crisis, burdens will fall disproportionately upon lower income countries, who have access to the fewest care resources to respond.^11^

Thinking ecologically allows the haematology community to identify better treatments now and will become indispensable in the coming years. The imminent upheaval of climate change upon haematology practice is profound and unsettling. Thinking in terms of ecosystems will be indispensable when familiar clinical presentations become transfigured by the ongoing climate emergency.

## AUTHOR CONTRIBUTIONS

**Stephen P. Hibbs:** Conceptualization; writing original draft; writing review editing.

## CONFLICT OF INTEREST STATEMENT

The author has no conflict of interest.

## FUNDING

S.P.H. is supported by a HARP doctoral research fellowship, funded by the Wellcome Trust (grant number 223500/Z/21/Z).

## Data Availability

Data sharing not applicable to this article as no datasets were generated or analyzed during the current study.
